# Survey of 17 elements, including rare earth elements, in chilled and non-chilled cauliflower cultivars

**DOI:** 10.1038/s41598-019-41946-z

**Published:** 2019-04-01

**Authors:** Andrzej Kalisz, Agnieszka Sękara, Sylwester Smoleń, Aneta Grabowska, Joanna Gil, Monika Komorowska, Edward Kunicki

**Affiliations:** 1 Department of Vegetable and Medicinal Plants, University of Agriculture in Kraków, 29 Listopada 54, 31-425 Kraków, Poland; 2 Unit of Plant Nutrition, Institute of Plant Biology and Biotechnology, University of Agriculture in Kraków, 29 Listopada 54, 31-425 Kraków, Poland

## Abstract

This study investigated if genetic diversity among cauliflower cultivars (white ‘Xenia’ F_1_, green ‘Vitaverde’ F_1_, purple ‘Graffiti’ F_1_, orange ‘Sunset’ F_1_, romanesco ‘Celio’ F_1_) and transplant chilling are reflected in the content of 17 elements in mature curds. Transplants at 40 days after sowing were exposed to 4 °C (chilling) and 18 °C (control) for 7 days and then planted in the field till harvest maturity. The lowest Ag, Al, Co, and Li contents were found in ‘Celio’ F_1_ cauliflower, which also had the highest Ba and Sr levels. Orange curds of ‘Sunset’ F_1_ were the richest in Al, and high in Li, Sc, and Sn. Chilling applied to the transplants increased Ag, Ba, Co, Sc, Sr, and Tb, and decreased the Y content of mature curds. Transplant chilling can permanently alter plant metabolism, and subsequently may affect the mineral composition of the curds.

## Introduction

Cauliflower (*Brassica oleracea* L. ssp. *botrytis*) is an important vegetable crop grown worldwide for its edible curds, being composed of undifferentiated shoot apices formed upon thick, hypertrophied, repeatedly branched terminals of the short, thick stem. It is well regarded for its high nutritional and low caloric value. The major bioactive compounds of white cauliflower are vitamins (C, E, B_1_, B_2_, B_3_, B_12_), phenolics, and dietary fiber, while the predominant glucosinolates in cauliflower are glucoiberin, sinigrin, and glucobrassicin^1^. White cauliflower has been reported to have higher K, P, Mg, Cu, Zn, and Ni or comparable Fe mineral content relative to white cabbage, whereas the curds have less Ca, Mn, and Se^2^. Element contents in Brassica vegetables vary among cultivars^3–5^, which is particularly relevant to cauliflower because, in addition to the large group of white-curded cultivars, breeding techniques have resulted in commercially available genotypes forming green (typical or pyramid-shape – so-called romanesco cauliflower), purple, and orange curds, with enhanced synthesis of chlorophylls, anthocyanins, and carotenoids, respectively^6^. Cauliflower genotypes show differences in the content of bioactive compounds^7^, as well as in the elemental composition^8,9^.

The literature indicates differentiation in the content of trace elements (TEs), including rare earth elements (REEs), in plant species^10,11^. Concerning the *Brassica* genus, Wen *et al*.^12^ found lower bioaccumulation of REEs in cabbage than in Chinese cabbage. Kučera *et al*.^13^ determined the concentration of REEs in two *B. oleracea* L. crops, cauliflower and kale, collected from a polluted region of Moravia, Czech Republic. They found a similar content of Sm, Yb, and Tb, and lower Ce and La content in cauliflower than in kale. Ekholm *et al*.^2^ observed a lower content of Al and Co in cauliflower relative to broccoli, while cauliflower curds had a 1.8-fold higher Co concentration than white cabbage heads. These data highlight the diverse accumulation of chemical elements within *B. oleracea* L. Nonetheless, published reports on cauliflower differentiation among cultivars are not numerous^8,9^.

Some of the TEs included in this report are essential for plant organisms in small amounts (Al, Co, Li, Sr, Ti), while others have no or weakly recognized functions in plants (Ag, Ba, Sb, Sn, Sc, Y, lanthanides [LAs]). The physiological role of these elements is unknown also in humans and animals^11,13^. Essential TEs are involved in controlling the structure and function of the plasma membrane and certain enzymes (Al, Ti), synthesis of chlorophylls and proteins, enhancement of stress resistance (Co, Ti), halophyte metabolism (Li), processes similar to that of calcium (Sr), and in photosynthesis (Ti)^11,14^. REEs are usually considered non-essential in plants, but there are several reports, including some contradictory opinions, on the physiological effect of REEs on membrane stabilization, plant enzyme activities, photosynthesis activity, chlorophyll content, proline biosynthesis, hormone effectiveness, growth, and water loss by plants^12,15,16^. Trace elements can act as supplements that load and reprogram the antioxidant system and lead to osmotic balance^17^. Although some TEs are essential, if their accumulation is excessive, potentially harmful effects on the plants can occur, but more dangerous is their entry into the food chain and deleterious impact on human health. People can be exposed to these elements because of contaminated environment components like food or water, which generate a serious safety problem in a global and local scale. An increased supply of trace elements to environment derives to a large extent from human activities, e.g. REE-based fertilizers are widely used to increase the yield and quality of crops particularly in China^18,19^. Availability of plant genotypes to accumulate trace elements will facilitate future breeding of crops toward reduced content of undesired elements.

Plant species and cultivars may differ in the uptake, translocation, accumulation, and utilization of chemical elements. Although these differences are under genetic control, their expression may be significantly altered in extreme environmental conditions, when various abiotic stresses act on the plants. Controlled application of low temperature, affecting plants’ metabolome, changes the chemical composition of plants and may refer to TEs, particularly those performing certain functions in plants’ physiological and metabolic processes. Such changes may also be permanent^20^. Therefore, the response of the plants to abiotic stress applied in the juvenile stage may lead to a metabolic predisposition, which causes differences in the accumulation of several elements in fully-matured edible plant parts.

Considering the information described above, we hypothesise that: 1) the concentrations of TEs in the cauliflower curds will be different in tested genotypes; and 2) low-temperature stress applied to the plants in the early stage of ontogeny will modify the chemical composition of the mature curds.

## Results

**Figure 1:**
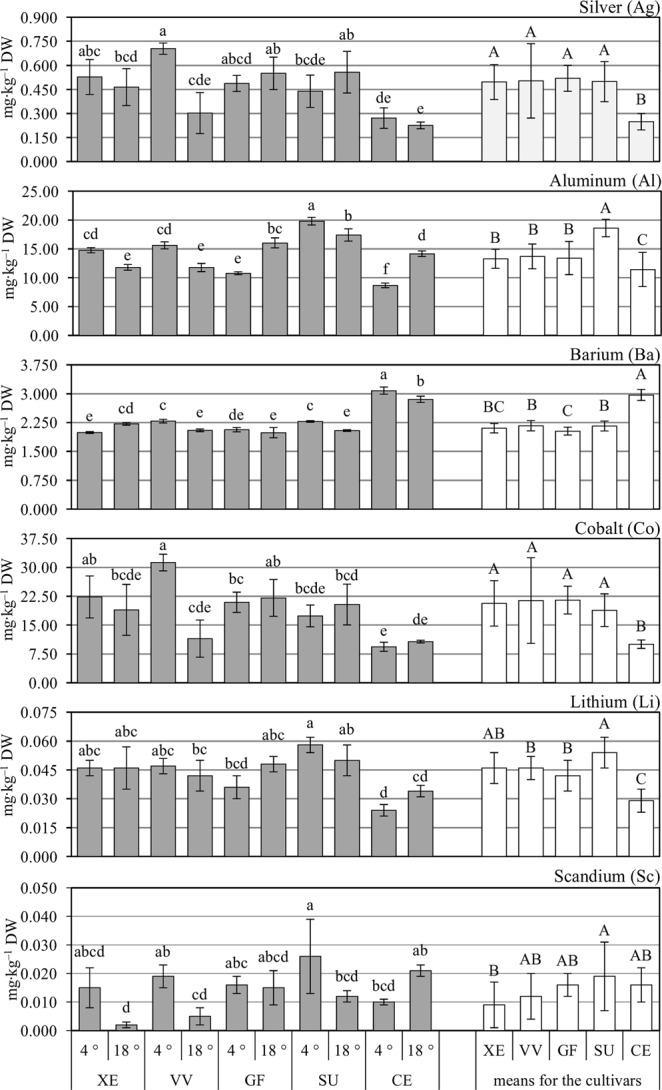
Content of Ag, Al, Ba, Co, Li, and Sc in cauliflower curds depending on the cultivar (CV: XE – white ‘Xenia’ F_1_, VV– green ‘Vitaverde’ F_1_, GF – purple ‘Graffiti’ F_1_, SU – orange ‘Sunset’ F_1_, CE – romanesco ‘Celio’ F_1_) and temperature (T: 4 °C, transplant chilling; 18 °C, control). Means followed by different capital letters for cultivar effects (CV; white columns), and lower-case letters for interaction effects (CV × T; grey columns) are significantly different at *p* ≤ 0.05, *n* = 4. No letter denotes no significant difference between means. Comparisons performed using Tukey’s honestly significant difference test. Error bars represent ± standard deviation (SD).

Among the cauliflower cultivars tested, the lowest contents of Ag, Al, Co, and Li were found in romanesco curds of ‘Celio’ F_1_ cultivar (Fig. 1). The highest content of Al was determined in curds of ‘Sunset’ F_1_. Orange curds of this cultivar contained 86.2% more Li than romanesco plants. The Ba content was highest in ‘Celio’ F_1_ compared to the other cultivars studied, but mainly in purple cauliflower (‘Graffiti’ F_1_), which accumulated 30% less Ba than ‘Celio’ F_1_. The Sc concentration was higher in cauliflower that formed orange curds (‘Sunset’ F_1_), but differences were significant only in comparison to the ‘Xenia’ F_1_ cultivar. Treating transplants with low temperature (4 °C) subsequently increased the average content of Ag, Ba, Co, and Sc in the curds, by 15.6%, 5.0%, 21.2%, and 54.5%, respectively; however, there was no effect of temperature on Al and Li (Tables 1 and 2). The results indicate that the contents of the elements analyzed were also controlled by interaction effects (Fig. 1, Table 2). The interaction effects for Ag and Co concentration were most apparent in ‘Vitaverde’ F_1_ cauliflower, with the average increase in these elements, due to an early low-temperature treatment, amounting to 132.7% and 172.4%, respectively. Interaction effects were most significant for Sc accumulation in ‘Vitaverde’ F_1_ and ‘Sunset’ F_1_ cultivars, where the low temperature increased the Sc content by 280.0% and 116.7%, respectively, compared to plants subjected to the control temperature (18 °C).

**Table 1:** Average content of chemical elements in mature cauliflower curds as influenced by low-temperature treatment of the transplants.

Symbol	Chemical elements content (mg∙kg^−1^ DW)	
	4 °C	18 °C
Ag	0.486 ± 0.159 A	0.421 ± 0.167 B
Al	13.910 ± 4.014	14.220 ± 2.409
Ba	2.340 ± 0.400 A	2.229 ± 0.337 B
Co	20.250 ± 7.830 A	16.710 ± 6.477 B
Li	0.042 ± 0.012	0.044 ± 0.009
Sc	0.017 ± 0.009 A	0.011 ± 0.007 B
Sm	0.521 ± 0.334	0.472 ± 0.369
Sn	0.336 ± 0.142	0.310 ± 0.148
Sr	6.010 ± 0.978 A	5.558 ± 0.897 B
Ti	1.823 ± 0.447	1.752 ± 0.391
Yb	0.032 ± 0.02	0.028 ± 0.016
Ce	7.650 ± 3.031	7.746 ± 3.946
Dy	0.024 ± 0.007	0.023 ± 0.008
La	0.033 ± 0.009	0.029 ± 0.005
Sb	1.188 ± 0.413	1.376 ± 0.482
Tb	0.029 ± 0.015 A	0.019 ± 0.008 B
Y	0.024 ± 0.013 B	0.035 ± 0.019 A

Means in a row followed by different letters are significantly different at *p* ≤ 0.05 according to Tukey’s HSD test, *n* = 4. No letter denotes no significant difference between means. Each value represents the mean ± standard deviation (SD).

**Table 2:** ANOVA results for the content of chemical elements in mature cauliflower curds as influenced by cultivar (CV: white ‘Xenia’ F_1_, green ‘Vitaverde’ F_1_, purple ‘Graffiti’ F_1_, orange ‘Sunset’ F_1_, romanesco ‘Celio’ F_1_) and temperature (T: 4 °C, transplant chilling; 18 °C, control), including the CV × T interaction, *n* = 4.

Symbol	Sources of variation		
	Cultivar (CV)	Temperature (T)	CV × T
Ag	***	*	***
Al	***	NS	***
Ba	***	***	***
Co	***	*	***
Li	***	NS	**
Sc	**	**	***
Sm	**	NS	*
Sn	**	NS	NS
Sr	***	***	***
Ti	***	NS	***
Yb	*	NS	*
Ce	NS	NS	NS
Dy	NS	NS	**
La	NS	NS	NS
Sb	NS	NS	NS
Tb	NS	*	NS
Y	NS	*	*

Level of significance: *p ≤ 0.05; **p ≤ 0.01; ***p ≤ 0.001; NS ‒ not significant. Elements have been arranged in two groups with respect to the significant effect of cultivar (CV) on their content, and then alphabetically.

**Figure 2:**
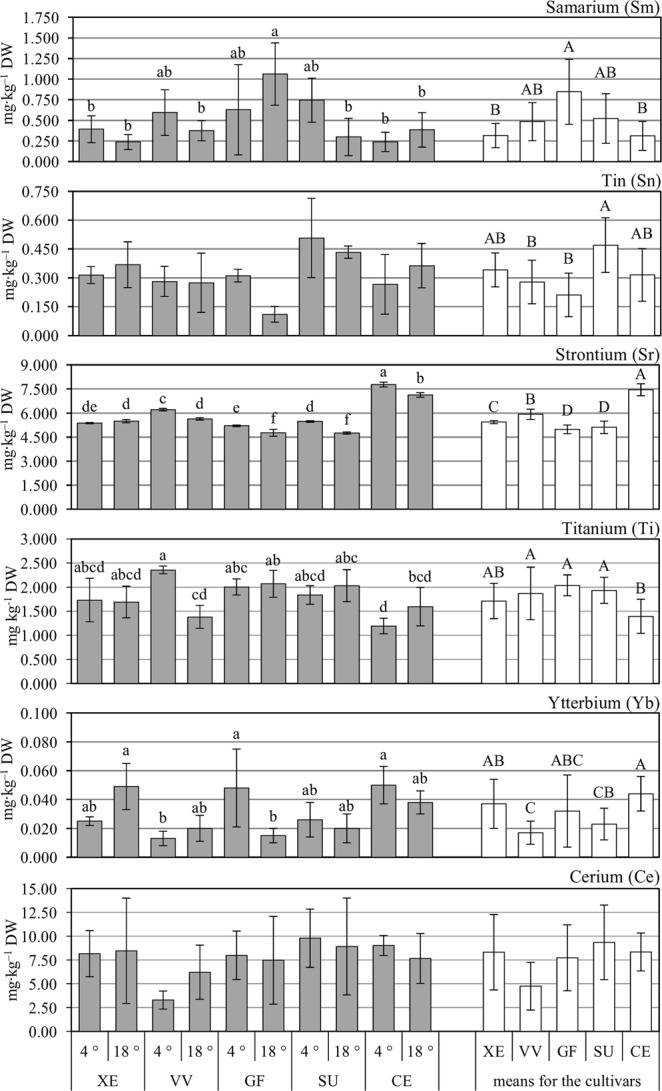
Content of Sm, Sn, Sr, Ti, Yb, and Ce in cauliflower curds depending on the cultivar (CV: XE – white ‘Xenia’ F_1_, VV– green ‘Vitaverde’ F_1_, GF – purple ‘Graffiti’ F_1_, SU – orange ‘Sunset’ F_1_, CE – romanesco ‘Celio’ F_1_) and temperature (T: 4 °C, transplant chilling; 18 °C, control). Means followed by different capital letters for cultivar effects (CV; white columns), and lower-case letters for interaction effects (CV × T; grey columns) are significantly different at *p* ≤ 0.05, *n* = 4. No letter denotes no significant difference between means. Comparisons performed using Tukey’s honestly significant difference test. Error bars represent ± standard deviation (SD).

The contents of Sm, Sn, Sr, Ti, and Yb were significantly different among cauliflower cultivars, but genotype did not play a role in regulation of the Ce level (Fig. 2). Sm contents were lowest in ‘Celio’ F_1_ and ‘Xenia’ F_1_ cauliflower, but only when compared with purple-curded ‘Graffiti’ F_1_ plants. The Sn concentration was higher in ‘Sunset’ F_1_ cauliflower, but differences were significant only relative to ‘Vitaverde’ F_1_ and ‘Graffiti’ F_1_ (green and purple morphotypes, respectively). The highest Sr content was found in ‘Celio’ F_1_ cauliflower, while significantly lower levels of this element were determined particularly in curds of ‘Graffiti’ F_1_ and ‘Sunset’ F_1_ cultivars, by around 33.1% and 31.4%, respectively. ‘Celio’ F_1_ had the highest amount of Yb, but only in comparison to ‘Vitaverde’ F_1_ and ‘Sunset’ F_1_ cultivars. Conversely, the romanesco ‘Celio’ F_1_ had significantly less Ti than the other cultivars (except ‘Xenia’ F_1_). Averages for the temperature effect alone showed that only the Sr content was significantly affected by transplant chilling, which caused an 8.1% increase in its level vs. the control plants (Table 1). The interaction effect influenced the accumulation of Sm, Sr, Ti, and Yb (Fig. 2, Table 2). In four of the five cultivars tested, the low temperature applied to the transplants increased the Sr level in the curds; the exception was ‘Xenia’ F_1_ cauliflower. A significant increase in Ti occurred in ‘Vitaverde’ F_1_ cauliflower chilled during the transplant stage (by 70.5%), while for chilled purple-curded plants (‘Graffiti’ F_1_), Yb was significantly increased (by 220.0%, on average).

**Figure 3:**
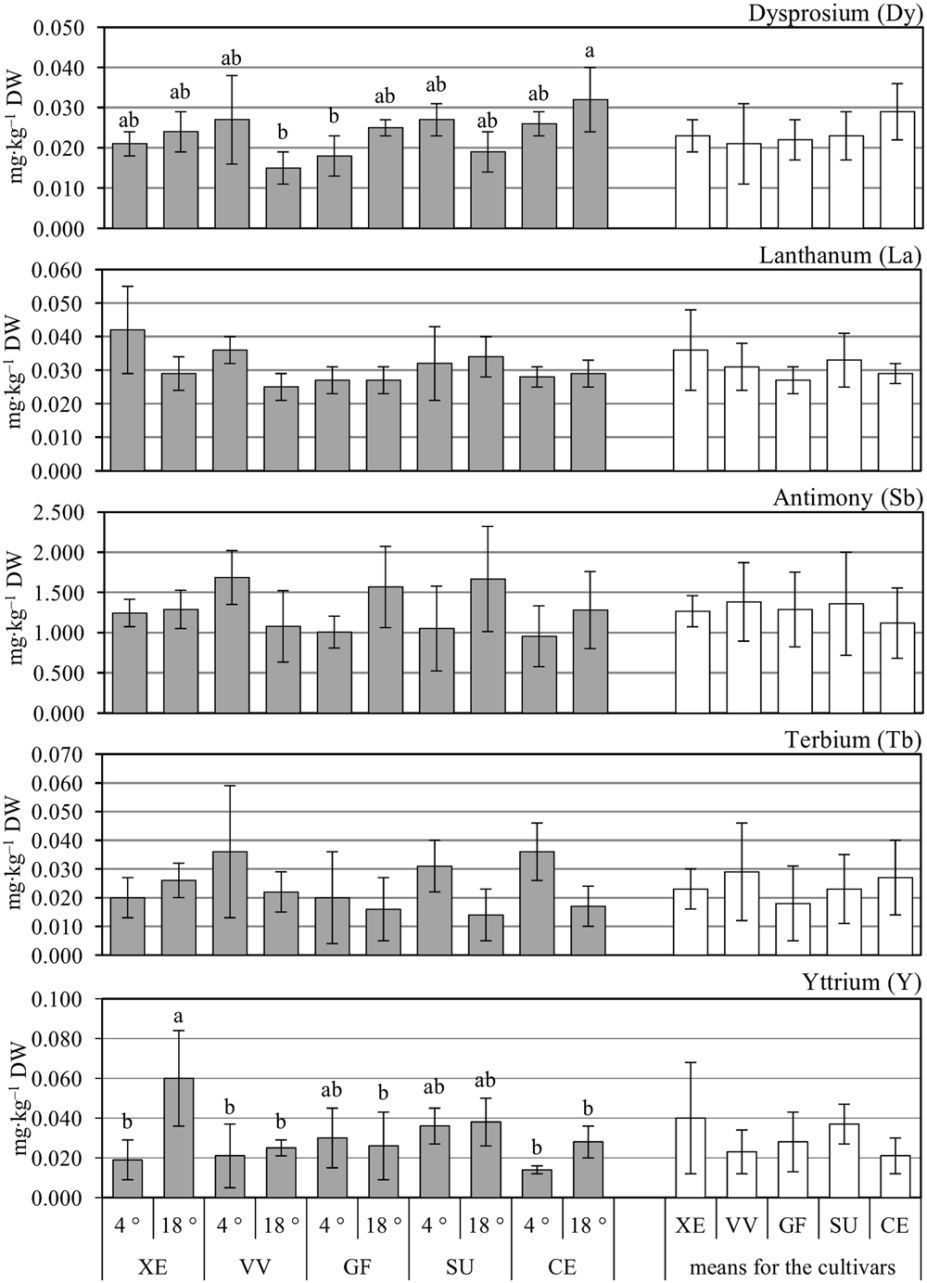
Content of Dy, La, Sb, Tb, and Y in cauliflower curds depending on the cultivar (CV: XE – white ‘Xenia’ F_1_, VV– green ‘Vitaverde’ F_1_, GF – purple ‘Graffiti’ F_1_, SU – orange ‘Sunset’ F_1_, CE – romanesco ‘Celio’ F_1_) and temperature (T: 4 °C, transplant chilling; 18 °C, control). Means followed by different capital letters for cultivar effects (CV; white columns), and lower-case letters for interaction effects (CV × T; grey columns) are significantly different at *p* ≤ 0.05, *n* = 4. No letter denotes no significant difference between means. Comparisons performed using Tukey’s honestly significant difference test. Error bars represent ± standard deviation (SD).

There were no significant effects of cauliflower genotype on the accumulation of Dy, La, Sb, Tb, and Y in the curds, with all cultivars displaying statistically similar levels of these elements (Fig. 3). No effect of transplant chilling on Dy, La, and Sb content was observed, but Tb was increased by 52.6%, on average (Table 1). Conversely, when low temperature was applied to the plants at the juvenile stage, the level of Y was decreased significantly compared to the control (by 31.4%). The interaction effects were important only for Dy and Y (Fig. 3, Table 2). Curds of the control ‘Xenia’ F_1_ plants had significantly more Y (by 215.8%, on average) than cauliflower plants chilled at the transplant stage. The greatest difference in Dy content was observed between control ‘Celio’ F_1_ plants, which had more Dy (by 113.3 and 77.8%, on average) than control ‘Vitaverde’ F_1_ and chilled ‘Graffiti’ F_1_ cauliflowers, respectively.

**Figure 4:**
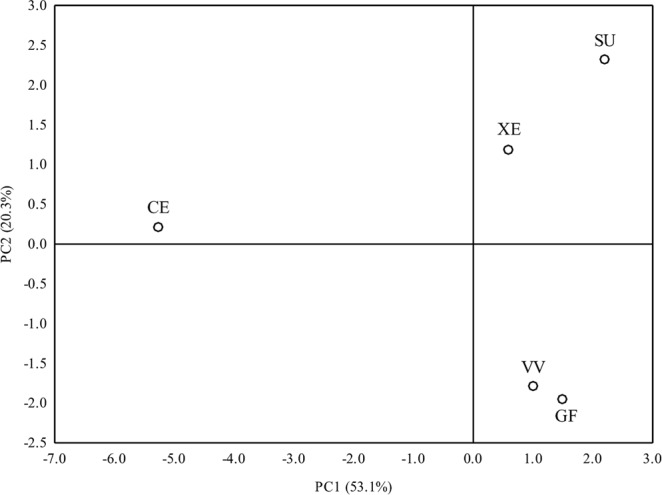
Ordination diagram obtained by principal component analysis (PCA) showing similarities among cauliflower genotypes (XE – white ‘Xenia’ F_1_, VV– green ‘Vitaverde’ F_1_, GF – purple ‘Graffiti’ F_1_, SU – orange ‘Sunset’ F_1_, CE – romanesco ‘Celio’ F_1_) according to the content of 17 elements tested in the curds.

PCA was done to investigate differences among cultivars in the accumulation of chemical elements (Fig. 4). The data revealed that PC1 and PC2 accounted for 73.4% of the total variance within the data set, contributing 53.1% and 20.3%, respectively. PC1 allowed differentiation between ‘Celio’ F_1_ (romanesco curds) and all other cauliflower genotypes tested. PC2 clustered ‘Vitaverde’ F_1_ and ‘Graffiti’ F_1_ cultivars on the negative side, while ‘Xenia’ F_1_ and ‘Sunset’ F_1_ cultivars were placed in the upper right quadrant (Fig. 4). Ag (0.979), Co (0.929), Li (0.911), Ti (0.931), Sb (0.907), and Al (0.684) contributed highly to PC1 but were inversely correlated with Ba (−0.966), Sr (−0.957), Dy (−0.943), and Yb (−0.727). PC2 was directly correlated with Sn (0.928), mainly, but also with Ce (0.763) and La (0.717). Given the positive loading of PC1 with the elements mentioned above, it is possible to conclude that the ‘Celio’ F_1_ cultivar is characterized by low Ag, Co, Li, Ti, Sb, and Al content but high levels of Ba, Sr, Dy, and Yb.

**Figure 5:**
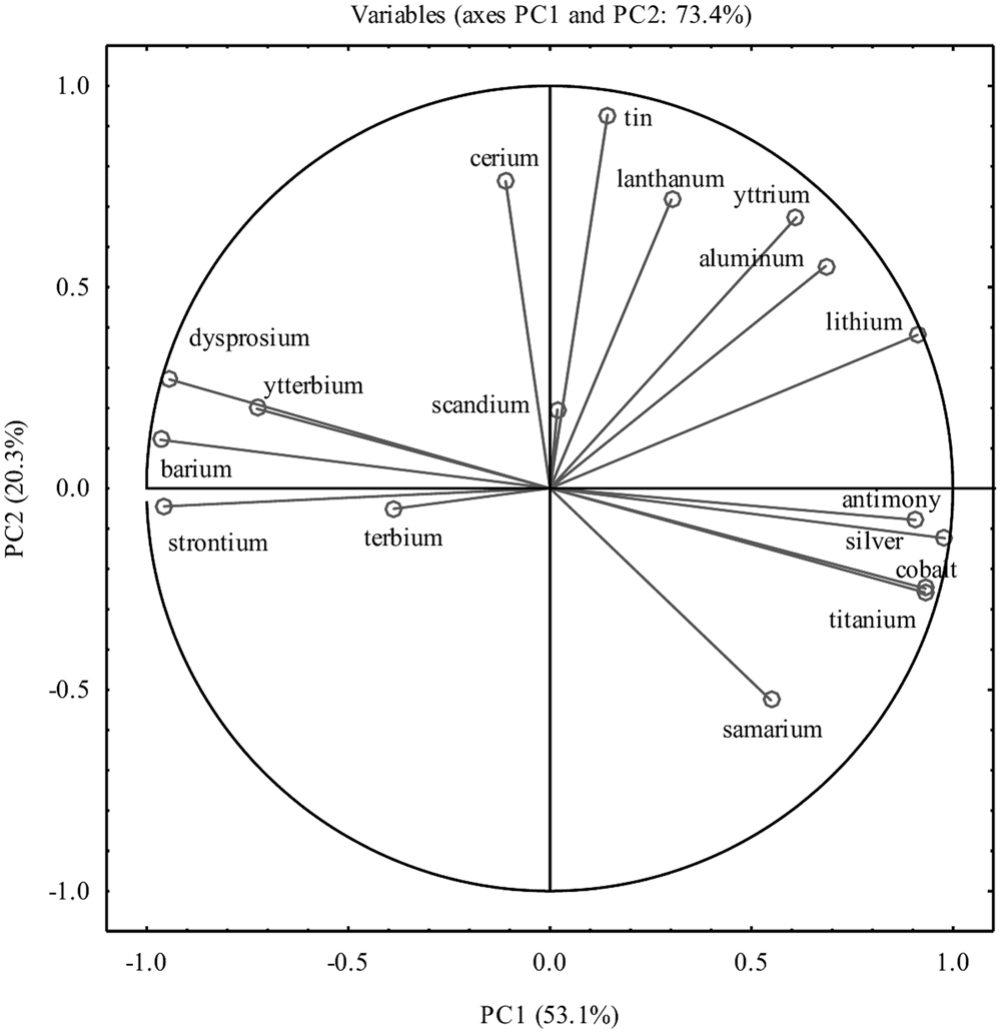
Integrated comparative bi-plot based on principal component analysis (PCA) showing the position of the variables projected in the plane as determined by the first two principal axes (73.4% explained variance).

There were some significant relationships among the chemical elements analyzed (Fig. 5). Inverse correlations were found between the contents of Ag and Ba, and Sr and Dy, whereas positive associations occurred between Ag and Co or Ti. Ba was negatively correlated with Co and Ti, but positive associations were observed for Ba and Sr or Dy. The Sr content had a significantly negative correlation with Co and Ti, and likewise for Dy and Co, and Ti and Sb. The latter was also negatively correlated with Yb.

Soil contents of trace elements, as well as pH and EC before planting and after harvest are presented in Table 3. Soil content of Al, Ba, Sm, Yb, Dy, La and Y increased significantly during growing season, while content of some other elements (Li, Sc, Sr, Ce, Sb, Tb) decreased. There were no significant changes for Co, Sn and Ti in the 0–30 cm deep soil layer. Soil samples collected after plant cultivation had lower pH values compared with the samples taken before experiment. On the other hand, soil electrical conductivity (EC) significantly increased in the soil during cauliflower growth.

**Table 3:** Content of trace elements, pH and EC in the 0–30 cm soil layer before plant cultivation and after plant harvest.

Element symbol	Before plant cultivation Mean (mg∙kg^−1^ DM of soil)	After plant harvest Mean (mg∙kg^−1^ DM of soil)
Ag	ND	ND
Al	917.47 ± 11.196 B	1160.00 ± 7.615 A
Ba	35.46 ± 0.346 B	53.83 ± 0.367 A
Co	1.665 ± 0.008	1.686 ± 0.013
Li	0.670 ± 0.017 A	0.577 ± 0.015 B
Sc	0.030 ± 0.001 A	0.027 ± 0.001 B
Sm	1.310 ± 0.010 B	1.530 ± 0.012 A
Sn	25.02 ± 1.508	25.79 ± 2.573
Sr	21.72 ± 0.383 A	11.81 ± 0.075 B
Ti	7.715 ± 0.017	7.764 ± 0.037
Yb	0.400 ± 0.002 B	0.434 ± 0.003 A
Ce	12.91 ± 0.064 A	12.51 ± 0.086 B
Dy	0.565 ± 0.003 B	0.684 ± 0.005 A
La	5.090 ± 0.014 B	5.609 ± 0.041 A
Sb	0.605 ± 0.008 A	0.419 ± 0.011 B
Tb	0.130 ± 0.004 A	0.111 ± 0.002 B
Y	5.235 ± 0.017 B	5.809 ± 0.041 A
pH in H_2_O	7.33 ± 0.01 A	7.03 ± 0.01 B
EC (mS∙cm^–1^)	0.10 ± 0.01 B	0.24 ± 0.01 A

Means in a row followed by different letters are significantly different at p ≤ 0.05 according to Student’s t test, *n* = 4. No letter denotes no significant difference between means. Each value represents the mean ± standard deviation (SD), ND ‒ not detected.

Available and reserve fraction of all elements were analyzed by extracting them from the soil with 1 M HCl.

## Discussion

### Reasons for diversity in TEs accumulation by plants

The bioavailability of chemical elements for plants depends on the physicochemical properties of the soil, other environmental conditions like microclimate, the plant genotype itself, and plant × environment interactions^11^. The entry of TEs, including REEs, into the biological environment is increasing due to different anthropogenic activities: from industry to extensive agriculture^21,22^. An example is application of REEs as fertilizers in East Asian countries, which might cause spreading of these metals in ecosystems^18,19^. Many studies have revealed that there is a large degree of variability in processes such as uptake, translocation, and accumulation of certain chemical elements in vegetable species, and even at the cultivar level^2,5,23,24^. These processes involve multiple molecular components including transporters, channels, chelators, and the genes that encode and regulate them^25^. It is well established that the accumulation of chemical elements in plants is controlled by one specific gene or group of genes; some studies have shown that the elemental composition of a plant tissue is highly plastic but tightly controlled by genes and gene × environment interactions^25^. Basically, trace elements can be classified in terms of the observed level of accumulation in plants as follows: group 1 involves elements with lack accumulation in the plants (e.g. Ba, Sc, Ti); group 2: elements showing slight accumulation (e.g. Li); group 3: elements showing medium accumulation (e.g. Ag, Co, Sr); group 4: elements which are intensively accumulated (e.g, Br, Cd)^11,26^. This classification, however, may change: components of variation in the elemental composition of plants, including physiological mechanisms and morphological features related to the genetic background, were described by Baligar *et al*.^27^. Briefly, the ability of a particular plant to gather certain elements is determined by root depth and morphology, retention of the elements in the roots, biomass production and shoot demand, internal transport in the plant, the presence of intracellular binding sites, vacuolar sequestration, and the activity of elemental transport through cell membranes^21,28^. Availability of elements to the plants depends on uptake processes and the transport of the elements to the above-ground parts. Additionally it may result from an inherently low element status of soil, low mobility of elements within soil, poor solubility of the given chemical form of the elements, production of exudates by plant roots mobilizing elements, or the soil-microbe-plant interactions^29^. Differences in the ability of the plant genotypes to access soil elements include differences in the surface area of contact between roots and soil and in the composition and amount of root exudates and interaction of roots with microorganisms, all of these resulting in differences in the chemistry and biology of the rhizosphere^30^.

### Genetic variability in the content of trace elements in cauliflower and other Brassicas

The cauliflower cultivars examined in the present experiment are phenotypically diverse, so planning the experiment, we believed that the chemical composition of the unique edible organs of cauliflower – curds – would be different. Only a few studies have focused on the accumulation of the TEs and REEs examined in this work in *Brassica* species^12^. Therefore, we screened cultivar effects within cauliflower plants for the accumulation of 17 elements in total (Ag, Al, Ba, Co, Li, Sn, Sr, Ti, Sb) including REEs (La, Ce, Sm, Tb, Dy, Yb, Sc, Y), filling a gap in the literature data. We found that accumulation of most of the chemical elements varied markedly between cauliflower genotypes, which resulted from the reasons described above and in the following sub-sections.

#### Group 1 element – Li

Plants belonging to the Brassicaceae family are not considered accumulators of Li, but some genetic differences have been documented; this element is present at a higher concentration in cabbage plants than in Brussels sprouts^10^. We found significant differentiation in Li content between some cauliflower cultivars of colored curds. Li can be connected to the metabolism of photosynthetic pigments. Although interactions between Li and photosynthetic pigments are unknown, Makus *et al*.^31^ noted that Li application reduced the leaf chlorophyll *a*:*b* ratio in spinach and mustard leaves.

#### Group 2 elements – Sr and Ba

Cultivar differences in accumulation of Sr and Ba in cauliflower curds were also tested. The Sr content in the plants seems to be lowest in grains (mean 1.5–2.5 mg·kg^−1^ dry weight [DW]), and highest in the leaves (45–74 mg·kg^−1^ DW^11^). Based on our results, cauliflower curds are weak accumulators of Sr. A large number of plants contain small but essential quantities of Ba in tissues (4–50 mg·kg^−1^ DW^32^); in this context, cauliflower can be counted among the low Ba-accumulating plants. It is interesting that in the group of cultivars tested, romanesco ‘Celio’ F_1_ presented the highest levels of Ba and Sr. Because Sr and Ba are homologous elements, the same absorption and transport mechanisms may be involved in their accumulation^33^. Some genotypic differentiation in these alkaline metals shown by Bibak *et al*.^10^ points to cabbage as a plant with a higher content of both of these elements than Brussels sprouts. Watanabe *et al*.^33^ found some differences in Sr and Ba concentration between commercial cultivars of bok choy (*Brassica rapa* var. *chinensis*) and komatsuna (*Brassica rapa* var. *perviridis*).

#### Group 3 elements – Sc and Y

Sc and Y are often included with the REEs because these two metals have similar properties to REEs, and they occur together with LAs in ores. In experiment, Sc occurred in cauliflower curds within the range documented by Sager^34^ as typical for green plants (0.012–0.261 mg·kg^−1^ DW). The concentration of Sc in plants is rather stable, and roots usually accumulate 10 times more Sc than leaves^35^. Our observations point to ‘Sunset’ F_1_ cauliflower as the cultivar accumulating this element more than ‘Xenia’ F_1_ cauliflower. There is a paucity of data on Sc content in vegetable crops, but differentiation in Sc between higher plant species is known^36^. Sager^34^ determined 0.004–0.805 mg Y·kg^−1^ DW in green plants, and this range is in agreement with our results. Y is accumulated mostly in the roots, then in the leaves, and least in the stem. We did not find any significant differentiation in Y concentration between cauliflower cultivars in the curds, but different plant species may accumulate this element to a different extent^37^.

#### Lanthanides (La, Ce, Sm, Tb, Dy, Yb)

Among the LAs investigated, so-called light rare elements (La, Ce, and Sm) are more basic, soluble, and mobile than heavy rare elements (Tb, Dy, and Yb). There are some reports on the stimulating impact of LAs on several processes in plants, but these elements have not yet been proved to be essential^11,18^. Ding *et al*.^38^ and Wen *et al*.^12^ observed accumulation of LAs in various plant parts, in the descending order of roots > leaves > stems > grains. From the current study, we can conclude that cauliflower curds are the organ with the least accumulation of LAs because REEs could be retained by roots, and only small portions reached other parts of the plants. Bibak *et al*.^10^ noticed a higher concentration of LAs in cabbage than in Brussels sprouts, resulting in genotypic differentiation within *B. oleracea* L. Wen *et al*.^12^ found higher contents of La, Ce, Sm, Tb, Dy, and Yb in Chinese cabbage in comparison to cabbage. Our results provide additional information, in that within the cauliflower cultivars, only Yb and Sm were significantly differentiated, and larger amounts of these elements were found in ‘Celio’ F_1_ and ‘Graffiti’ F_1_ cultivars, respectively.

#### Group 4 element – Ti

Ti is considered as beneficial for plant growth, and its level in plants varies considerably from 1.0 to 578.0 mg·kg^−1^ DW^14^; the content in the cauliflower curds seems to be at the lower end of this range. Smaller amounts of Ti were found in ‘Celio’ F_1_ plants compared to most other cauliflower cultivars, which indicates that the content of this element is dependent on the genetic specificity of plants, which was shown for different vegetable crops species by Lyu *et al*.^14^.

#### Group 9 element – Co

In plants, the Co concentration ranges from 0.1 to 10 mg·kg^−1^ DW^35^; therefore, the cauliflower curds had an elevated Co content. Ekholm *et al*.^2^ showed different levels of Co between *Brassica* crops (cauliflower, broccoli, and cabbage); of the cauliflower cultivars tested, ‘Celio’ F_1_ had the lowest content of that element, which indicates that uptake and distribution of Co is also cultivar-dependent.

#### Group 11 element – Ag

Kabata-Pendias^11^ described that Ag bioaccumulation decreased according to the order of shoot > root and the plant concentrations ranged from 0.03–0.5 mg∙kg^–1^ DW (the Ag concentration in cauliflower curds was at the top of this range). Ag can substitute for K^+^ sites in membranes and, thus, inhibit the absorption of other cations by roots. There is some evidence that Ag nanoparticles and AgNO_3_ treatment increased the total carotenoids in *Solanum tuberosum* explants^39^, however, the mechanism of Ag involvement in the metabolism of photosynthetic pigments needs future research. The current data conveyed a significantly higher Ag level in orange ‘Sunset’ F_1_ cauliflower than the other cultivars studied.

#### Group 13 element – Al

The average Al level in plant tissues is about 0.2 mg·kg^−1^ DW. Miller-Cebert *et al*.^40^ did not observe significant differences among *Brassica napus* cultivars; however, canola accumulated much more Al than all the other crops tested (cabbage, collard, and kale). Low Al levels can affect plant growth beneficially, especially Al-tolerant plant species. The Al tolerance of 11 species in six genera of the Brassicaceae indicates highly significant differences among them^41^. Cauliflower plants seem to accumulate Al at a high level, although tolerance/sensitivity of the cultivars investigated to Al requires future investigations. The high Al level in orange ‘Sunset’ F_1_ cauliflower indicates the involvement of this element in photosynthetic pigment metabolism. This observation concurs with Lazarević *et al*.^42^ who demonstrated that short exposure and/or small Al concentrations have a positive effect on the carotenoid pigments of potato.

#### Group 14 element – Sn

Sn may have an essential role at very low concentrations^43^. Kabata-Pendias^11^ found that Sn exists in crops at <0.1 mg·kg^−1^ DW. Sn shows low availability in soil and is not found in all plants. In comparison, we determined a high amount of Sn in cauliflower curds, especially in the ‘Sunset’ F_1_ cultivar. This shows that a specific cultivar may provide significant contributions to the intake of Sn.

#### Group 15 element – Sb

Another element, Sb, is considered a non-essential metal; it is relatively easily absorbed by plant roots when it is present in soluble forms. Hammel *et al*.^44^ examined Sb transfer into 19 crop species and detected 0.09 mg Sb·kg^−1^ DW in grains and other storage organs, whereas the maximum Sb concentrations in shoots and leaves were 0.34 and 2.20 mg·kg^−1^ DW, respectively. Hence, the Sb concentration in cauliflower curds was within cited limits. However, we did not find any effect of cultivar on the level of Sb in cauliflower, but genotypic variability exists among edible plants species^45^.

### TEs content in cauliflower curds as affected by transplant chilling

The present results indicate that plant metabolism may be changed due to chilling, which was reflected in an altered concentration of some chemical elements in mature cauliflower curds compared to that in untreated plants. Notably, chilling caused a significant increase in the content of several elements: Ag, Ba, Co, Sc, Sr, and Tb. Among them, Co is considered as essential in small amounts, participating in several physiological processes occurring in plants, particularly in N fixation by legumes^11^. Although the toxic effect of Ag on plants is well documented^46^, the physiological role of this chemical element is unknown, thus it is difficult to explain an increase of Ag content in chilled plants. Both Sr and Ba increased as a result of the chilling treatment of plants. Sr has chemical properties similar to Ca, and it can replace Ca, but it is not essential for plant growth^11^. Williams & David^47^ showed that the proportion of Ca to Sr in plant material is similar to that in a water extract of the soil; this suggests that these two ions are taken up by plants through the soil solution, without any marked discrimination between them. Ba is physiologically inactive in plants under normal circumstances, and its soluble salts are highly toxic; however, Ba is also a competitor to Ca and Sr, and can be taken up even preferentially by plants in comparison to Sr^33,48^. As was observed in an earlier study^9^, chilling of transplants causes an increase in Ca concentration in cauliflower curds. Perhaps these relationships explain the higher accumulation of Sr and Ba in chilled cauliflower.

Transplant chilling increased the accumulation of some REEs, namely Sc and Tb, in the curds of cauliflower, but decreased the content of Y, often considered as an REE. The remaining REEs were not affected by chilling of juvenile plants. Pang *et al*.^18^ described some effects of REEs, applied at low concentration, in mitigating abiotic stress of plants and supporting their antioxidant potential. Hu *et al*.^49^ in their review discussed the physiological interaction of REEs with Ca, effects on the structure and function of cytoplasm membranes, photosynthesis, hormone metabolism, enzyme activity, and water use efficiency. Although our data do not confirm any physiological role of some REEs, a relationship of Sc, Tb, and Y content with some physiological processes occurring in the plants cannot be excluded.

Chilling causes immediate changes in the plant organism, associated with substantial changes in metabolic pathways, including increased activity in the detoxification of reactive oxygen species^50^. Several non-enzymatic, as well as enzymatic, compounds, and even some chemical elements, present in plant tissues are reported to confer tolerance against abiotic stresses^11,16,18,51,52^. In many plant species, a period of mild exposure to stress stimulus may prime a plant against future stress or promote an acclimated state that may persist until a subsequent exposure^53^. The primed state is maintained over several days to weeks, implying that plants have some type of stress memory; after that, some stress-induced modifications revert to the initial level once the stress factor is removed, but some alterations in plant metabolism seem to be stable and more long-lasting^20,54^.

One possible manifestation of somatic stress memory is modified plant growth and metabolism resulted from epigenetic switch^54^, altering the uptake, distribution, and accumulation of chemical elements. This was observed by Sękara *et al*.^55^ who showed that chilling of transplants affected the levels of mineral nutrients in the fruits of previously stressed eggplants. As we noticed, chilling of the transplants affects the content of some chemical elements in mature curds of cauliflower, which is an evidence of long-lasting alterations in plant metabolism, but the mechanism by which accumulation of the elements is changed may be an interesting target for future research. Plant acclimation can be indicated by a reduced growth early in chilling period followed by higher growth rate after exposure to moderate chilling^56^. We think that faster growth of acclimated plants in the next stages of ontogeny increases shoot demands for elements, and together with higher water uptake and root hydraulic conductance of chilled plants^57^ it may result in greater uptake of the trace elements, including REEs, from the soil to plant organs. However, not only uptake processes are important, because maintaining the primed state requires the allocation of resources^54^. Different transport systems can be used by plants to allocate specific elements from roots to shoots and distribute between aboveground tissues of particular morphology and functions^58^. Thus, final accumulation of elements in plant tissues may be a result of their direct involvement in acclimatization processes^18,49^, distribution and dilution in the tissues on the subsequent stages of ontogeny as well as may be linked to interactions between nutrients and tested elements^59^.

### Changes in elements content in the soil during growing season

The interaction of geochemical, climatic and anthropogenic factors has superior influence on the bioavailability of trace elements in the soil environment during plant cultivation. As a consequence, the degree of bioaccumulation of trace elements by plants changes. Short- and long-term fluctuations in bioavailability of trace elements are commonly observed in the soil environment^26^. Short-term fluctuations include differentiated bioavailability of trace elements in soil during crops growth. They depend on physical, chemical, biochemical and microbiological soil factors (soil texture, pH, EC, Eh, CEC, SOM content etc.) as well as the applied fertilization (mineral and organic) and the course of climatic conditions (amount and distribution of rainfall and soil temperature). The physiological processes of plant roots, e.g. root exudations, have a large impact on short-term fluctuations in bioavailability of trace elements in soil^11,26^. In the studies of Wen *et al*.^12^, REEs fertilizer was used in cultivation of cucumber, tomato, Chinese cabbage, cabbage, radish and kidney bean. On the basis of the results, the authors did not recommend fertilizing with REEs due to the significant accumulation of Ce, Dy, Er, Eu, Gd, Ho, La, Lu, Ne, Pr, Sm, Tb, Tm, Y, Yb in vegetable crops – from bioavailable soil resources. The significant pH decrease and increase in soil EC after cultivation observed in our research was associated with applied mineral fertilization, root exudates, especially organic acids, as well as microbiological destruction of organic matter^60^. With lowering of pH the availability of most elements increased in line with vegetation progress.

Curds of cauliflower absorbed all the elements analyzed within this study, i.e. Ag, Al, Ba, Co, Li, Sc, Sm, Sn, Sr, Ti, Yb, Ce, Dy, La, Sb, Tb, Y (Figs 1–3). As a consequence, a significant amount of TEs was harvested from the field with the curds (Tables S1–S3). It should be emphasized that total uptake of the elements by the cauliflower showed generally similar relationships with the experimental factors as the element contents in curds expressed in mg∙kg^–1^ DW. In the soil, some of the elements showed a negative balance (Li, Sc, Sr, Ce, Sb, Tb), parts of them positive balance (Al, Ba, Sm, Yb, Dy, La and Y) while Co, Sn and Ti showed no significant differences in their content before and after cultivation of plants (zero balance) – Table 3. For elements with a negative balance in the soil after cultivation, their uptake by cauliflower plants was greater than the changes in the degree of bioavailability (short-term fluctuations). That could be due to the active bioaccumulation by cauliflower root system. In the case of elements with a positive balance in soil, the uptake and absorbing them by cauliflower curds from the soil was lower than the increase in the degree of their bioavailability resulting from the soil processes or their introduction to soil with fertilizers. On the other hand, in the case of elements with zero balance, the plant uptake was balanced by the factors discussed above.

Application of organic, mineral and mineral-organic fertilizers, which contain the ballast trace elements and rare earth elements leads to enriching the soil in these two groups of elements^61–63^. Depending on the content of particular elements in fertilizers, their inflow to the soil may be equal to or greater than the degree of their uptake and removal from the soil with the plant yield. This can be a reason for the positive balance of Al, Ba, Sm, Yb, Dy, La and Y as well as zero balance of Co, Sn and Ti. In the present experiment, the available and reserve fraction of all seventeen elements was analyzed through extraction with 1 M HCl solution from the soil samples before and after cultivation of cauliflower. Therefore, the content of potentially available and readily soluble elements was monitored, not their total content in the soil. The positive or zero balance of TEs/REEs was probably the result of the supply with fertilizers and/or processes biochemical processes in the rhizosphere that occurred during cultivation of the plants.

### Chemical elements in the soil-plant interface

Final element concentrations in cauliflower curds depended also on plant growth and biomass formation, root morphology as well as on root activity involving rhizosphere interactions with soil microflora^64^. Bacteria may influence elements uptake through: increase of root surface area and hair production, increase element availability, and/or increase soluble element transfer from the rhizosphere to the shoots^65^. The influence of bacterial activity in rhizosphere on elements solubility has been reviewed by Gadd^66^. Microbes can modify elements mobility and bioavailability through several mechanisms: the release of chelating agents (e.g. organic acids, phenolic compounds, siderophores) and acidification or redox changes in the rhizosphere^67,68^. Sessitsch *et al*.^65^ claimed that mobilization of TEs and REEs by microbes can be achieved by acidification, chelation and ligand-induced dissolution. Plant uptake of REEs and other insoluble elements seemed to be linked to such ability of a plant to access a certain element pool in soils. Thus final elemental composition of the genotypes is strongly influenced by plant-enhanced mobilization of the elements in the rhizosphere.

## Conclusions

The study demonstrated variability in the accumulation of most of the elements analyzed among cauliflower cultivars. The ‘Celio’ F_1_ cultivar presented the lowest Ag, Al, Co, and Li concentrations, and the highest levels of Ba and Sr. These elements, not taken in large quantities by ‘Celio’ F_1_, belongs to alkali metals (Li), alkaline earth metals (Ba, Sr), and transition metals (Co, Ag), the only exception in this case is Al. ‘Sunset’ F_1_ cauliflower tended to accumulate more Al, Li, Sc, and Sn than the other cultivars. The ‘Graffiti’ F_1_ plants contained more Sm than white and ‘Celio’ F_1_ genotype. Complex activity of cauliflower rhizosphere affected the availability and uptake of investigated elements, reflected by positive, negative or zero balance of their content in the soil. Moreover, the xylem transport via root and shoot and distribution between leaves and growing curds influenced the final content in harvestable parts, highly genotype-depended. REEs and Ti form almost insoluble binding forms and they are poorly available to the plants. Therefore the availability of REEs and Ti may be affected by individual genotypes ability to modify rhizosphere biochemistry. The final level in cauliflower curds of elements with high mobility in the soil (e.g. Li) depended on internal physiology of ions transport, also differentiated between genotypes. Low-temperature treatment induced marked changes in the content of the elements in the cauliflower curds, increasing the levels of Ag, Ba, Co, Sc, Sr, and Tb, and decreasing the concentration of Y. Mechanisms by which the trace elements mediate or are mediated by abiotic stress are substantial but are not well known. The effectiveness of the antioxidant mechanism in plants depends on the intensity and duration of the stress factor. Trace elements are able to enhance the antioxidant system and support osmotic balance. Differences in TEs accumulation were noticeable in the mature cauliflower curds, long after the treatment of low temperature, which should be considered as plant stress memory.

## Methods

### Plant material and chilling treatment

Five cauliflower (*B. oleracea* L. ssp. *botrytis*) cultivars: white ‘Xenia’ F_1_ (Enza Zaden), green ‘Vitaverde’ F_1_ (Rijk Zwaan), purple ‘Graffiti’ F_1_ (Syngenta Seeds), orange ‘Sunset’ F_1_ (Clause Vegetable Seeds), and green/pyramidal romanesco ‘Celio’ F_1_ (Clause Vegetable Seeds) were examined. The seeds were germinated and grown for 40 days at optimal temperature (24 ± 2 °C) until emergence (4 days after sowing), and then placed in a greenhouse (18/15 ± 2 °C day/night). Afterward, transplants were transferred to a vegetative growth chamber set at 4 °C (day/night) and, in the case of the controls, to a chamber set at 18 °C, for 7 days. In all instances, the growth chambers were maintained at 75% relative humidity and a 14-h photoperiod at a photosynthetic photon flux density of 300 μmol·m^−2^·s^−1^ (Sunmaster LM 400 W U46 CDX metal halide lamps, Venture Lighting Europe Ltd, Rickmansworth, UK).

During experiments, conducted in 2013 and 2014, transplants were grown in 96-cell black trays (single cell volume of 53 cm^3^), filled with standard peat substrate (Klasman TS2, Klasmann-Deilmann GmbH, Geeste, Germany). Plants were fertilized twice with Kristalon Green liquid fertilizer (18% N, 18% P_2_O_5_, 18% K_2_O, 3% MgO, 2% S) from Yara International ASA, Poland, at a dose of 10 g·dm^−3^ water, 3–4 weeks after sowing, and only once with 98.5% ammonium molybdate ((NH_4_)_6_Mo_7_O_24_·4H_2_O; POCH SA, Poland), applied at the end of transplant production, at a dose of 1 g·dm^−3^ water.

### Field trials

The field trials were conducted at the experimental field of the University of Agriculture in Kraków, southern Poland (50°04′N, 19°51′E). According to Köppen’s classification, the climate of the region is humid continental (Dfb). Plots were established in the middle of April. After transplanting, they were covered with nonwoven fleece (Agryl PP, weight 19 g·m^−2^). Nonwoven covers were removed from the plants on about 10 May in both years.

The experimental design used to evaluate cauliflower chemical composition was a split-block (strip-plot); temperature was the horizontal-strip plots, and cultivar was the vertical factor (vertical-strip plots), with three replications (blocks). Cultivars were randomly assigned to these strips within each replication. Plant spacing was 50 × 45 cm, and a single plot with an area of 9 m^2^ contained 30 plants (plants for sampling plus the two outermost rows to eliminate border effects). The soil type was a Fluvic Cambisol (Humic). Soil pH (H_2_O) was 6.69, salinity was 0.50 g NaCl per cubic decimeter of soil, and organic matter was 2.76%. Tillage, irrigation, fertilization, weed management, and plant protection were conducted in agreement with the requirements of good horticultural practice. Fertilizers were applied based on soil test analyses, to achieve 140 mg N, 60 mg P, 200 mg K, 70 mg Mg, and 1500 mg Ca in 1 dm^3^ soil. The following fertilizers were applied to the soil before transplanting: 50% of the N dose: nitrochalk (13.5% N–NO_3_, 13.5% N–NH_4_, 2.0% CaO, 4.0% MgO) from Grupa Azoty SA (Poland), and twice during plant vegetation (25% dose of N per application) as calcium nitrate (14.5% NO_3_, 1.0% NH_4_, 19.0% Ca; CalciNit, Yara International ASA, Poland). P was applied as single superphosphate (19% P_2_O_5_, 25% CaO; 32% SO_3_; Siarkopol, Poland), and K as potassium chloride (60% K_2_O; Luvena SA, Poland). The Ca source was fodder chalk (Jaro SA, Poland) containing 93% Ca in the form of calcite (CaCO_3_). Borax (Na_2_B_4_O_7_·10H_2_O; Brinkman, Poland), containing 11.3% B, was applied at a dose of 15 kg·ha^−1^.

### Plant sampling and analyses

We investigated the content of Ag, Al, Ba, Co, Li, Sn, Sr, Ti, Sb, and all rare-earth elements. In the manuscript we showed only REEs successfully detected by ICP-OES (La, Ce, Sm, Tb, Dy, Yb, Sc, Y). Selected elements represent various groups of periodic table and they belong to different groups in relation to the accumulation availability by plants. Curds were harvested from 7 June to 1 July 2013 and from 23 June to 18 July 2014. Plant samples were collected during full harvest, and involved matured and healthy-looking curds, characteristic for each cultivar. Curds were cut into pieces and dried at 70 °C in a dryer with forced air circulation. Then, the plant material was ground into a fine and non-fibrous powder using a Pulverisette 14 ball mill (Fritsch GmbH, Germany) with a 0.5-mm sieve. Next, 0.5 g samples were placed in to 55 ml TFM vessels and were mineralized in 10 ml 65% super pure HNO_3_ (Merck no. 100443.2500) in a Mars 5 Xpress (CEM, USA) microwave digestion system^69^. The following mineralization procedure was applied: 15 min. time needed to achieve a temperature of 200 °C and 20 minutes maintaining this temperature. After cooling, the samples were quantitatively transferred to 25 ml graduated flasks with redistilled water. Contents of mentioned elements were determined using a high-dispersion inductively coupled plasma optical emission spectrometer (ICP-OES; Prodigy Teledyne Leeman Labs, USA)^70^. The spectrometer was calibrated using Merck’s ICP multi-element standard no. VI and no. XVI as well as Inorganic Ventures ICP (single element standard of Sn and multi-element of Rare69 element group). Each sample was measured four times. During the analysis of elements, the following parameters of the ICP-OES spectrometer were used. For an equipment: torch Dual View “3 slot” (quartz); normal slit (40 × 100 µm); type of nebulizer “High Solids Concentric Sea Spray”; spray chamber “Cyclonic with Knock-Out” (glass) 50 ml; entry tubes “2-Stop PCV Tubes 12’ Ø 0.76 mm” as well as exit tubes “2-Stop PCV Tubes 12’ Ø 1.14 mm”. For an operating parameters: RF Power 1.4 kW; pruge of optics “Low”; pump flow rate 1.4 ml·min^−1^; gas argon flow rates “Coolant” 18 dm^3^·min^−1^, “Auxiliary” 0.9 dm^3^·min^−1^, “Nebulizer” 35 Psi. For an analytical settings: time of uptake 60 s (adjusted to tubes length); standard uptake without using option “Fast uptake”; Integration no. 2; 20 s integration time of Axial position plasma observation for analysis of light on the camera (detector) emitted by elements in plasma. The elements were measured with using the best preferred analytical line (in nm): Ag 328.068, Al 396.152, Ba 233.527, Ce 413.765, Co 228.615, Dy 364.540, La 408.672, Li 670.784, Sb 206.833, Sc 361.383, Sm 359.260, Sn 189.991, Sr 407.771, Tb 350.917, Ti 334.941, Y 371.030, Yb 328.937.

We also calculated the mean of total trace elements concentration in cauliflower curds based on the average weight of the curds, the dry weight content and the concentration of elements in the dry weight (Tables 1S–3S) which reflects plant accumulation capacity.

### Soil sampling and analyses

Representative soil samples (3 samples per plot ×2 transplant chilling plots ×5 cultivar subplots) were collected from the 0–30 cm layer using a soil drill (3 cm diameter). Samples were taken from the plots before establishing experiment (mid-April) and after plant harvest (July). In the laboratory, quadruplicates were made for each sample. All soil ICP-OES analyses were performed by Prodigy Teledyne Leeman Labs (USA). Ag, Al, Ba, Co, Li, Sc, Sm, Sn, Sr, Ti, Yb, Ce, Dy, La, Sb, Tb, and Y were extracted with 1 M HCl^71^. During soil analysis, the same setting of the ICP-OES spectrometer was used as for the analysis of plant samples.

### Statistical analyses

All experimental data were analyzed using Statistica software, version 12.5 (StatSoft Inc., USA). Significant differences were determined by analysis of variance (ANOVA), followed by Tukey’s honestly significant difference post hoc test. Effects of cultivar (CV), temperature (T), and their interaction (CV × T) were evaluated at three levels of significance: *p* ≤ 0.05 (*), *p* ≤ 0.01 (**), and *p* ≤ 0.001 (***). Means were separated into homogeneous groups at *p* ≤ 0.05. Elements were arranged in two groups, concerning a significant effect of the cultivar (CV) on their content, and then alphabetically. All results have been presented as the mean of quadruplicates ± standard deviation (SD). Data were pooled over two study years. Standardized data (average values) were evaluated by principal component analysis (PCA) in Statistica software, to determine similarity in the chemical profile of the cauliflower cultivars tested. The eigenvalues were 9.02, 3.46, 2.57, and 1.70 for the following principal components (PCs). PC1 and PC2 described 73.4% of the total variance and were considered for discussion. Correlations between the chemical elements tested were studied among multidimensional datasets, through PCA. Soil data were compared by paired Student’s t-test with Statistica at *p* ≤ 0.05.

## Supplementary information

Supplemetary tables

## Data Availability

The basic data that support the findings of this study are available at http://www.nature.com/srep.
