# Supplementary material for: Survey of 17 elements, including rare earth elements, in chilled and non-chilled cauliflower cultivars
Source: Sci Rep. 2019 Apr 1;9:5416. doi: 10.1038/s41598-019-41946-z (PMC6443738; doi:10.1038/s41598-019-41946-z)
Supplement: Supplementary file 1 — Supplemetary tables [file 41598_2019_41946_MOESM1_ESM.pdf]

Supplementary Information

Survey of 17 elements, including rare earth elements, in chilled and non-chilled cauliflower cultivars

Andrzej Kalisz[1*], Agnieszka Sękara[1], Sylwester Smoleń[2], Aneta Grabowska[1], Joanna Gil[1], Monika Komorowska[1] & Edward Kunicki[1]

[1] Department of Vegetable and Medicinal Plants, University of Agriculture in Kraków, 29 Listopada 54, 31-425 Kraków, Poland

[2] Unit of Plant Nutrition, Institute of Plant Biology and Biotechnology, University of Agriculture in Kraków, 29 Listopada 54, 31-425 Kraków, Poland

[*] Correspondence and requests for materials should be addressed to A.K. (email: andrzej.kalisz@urk.edu.pl)

Table 1S. Uptake of Ag, Al, Ba, Co, Li, and Sc by cauliflower plants (mg per curds) as affected by cultivar and temperature treatment.

Cultivar	Temperature (° C)	Ag	Al	Ba	Co	Li	Sc
Xenia F_1	4	0.0337 ± 0.0061 ab	0.9456 ± 0.0178 a	0.1277 ± 0.0035 ab	1.4235 ± 0.3115 ab	0.00293 ± 0.00025 a	0.00094 ± 0.00050 ab
	18	0.0293 ± 0.0074 bc	0.7413 ± 0.0439 b	0.1393 ± 0.0036 a	1.1906 ± 0.4088 bc	0.00291 ± 0.00064 a	0.00120 ± 0.00000 e
Vitaverde F_1	4	0.0404 ± 0.0035 a	0.8920 ± 0.0303 a	0.1309 ± 0.0085 ab	1.7895 ± 0.1542 a	0.00269 ± 0.00036 ab	0.00111 ± 0.00024 a
	18	0.0167 ± 0.0065 def	0.6530 ± 0.0411 c	0.1138 ± 0.0060 c	0.6322 ± 0.2475 de	0.00235 ± 0.00050 abc	0.00030 ± 0.00018 de
Graffiti F_1	4	0.0209 ± 0.0020 cde	0.4610 ± 0.0314 d	0.0885 ± 0.0071 d	0.8939 ± 0.0953 cd	0.00155 ± 0.00030 de	0.00066 ± 0.00009 abcde
	18	0.0222 ± 0.0038 cd	0.6456 ± 0.0327 c	0.0800 ± 0.0050 de	0.8874 ± 0.1848 cd	0.00193 ± 0.00018 bcd	0.00059 ± 0.00022 abcde
Sunset F_1	4	0.0131 ± 0.0027 def	0.5940 ± 0.0187 c	0.0684 ± 0.0024 e	0.5204 ± 0.0720 de	0.00175 ± 0.00014 cde	0.00079 ± 0.00040 abcd
	18	0.0154 ± 0.0040 def	0.4795 ± 0.0335 d	0.0562 ± 0.0010 f	0.5616 ± 0.1518 de	0.00137 ± 0.00020 de	0.00032 ± 0.00004 cde
Celio F_1	4	0.0110 ± 0.0027 ef	0.3489 ± 0.0168 e	0.1240 ± 0.0029 bc	0.3761 ± 0.0425 e	0.00097 ± 0.00010 e	0.00040 ± 0.00003 bcde
	18	0.0098 ± 0.0010 f	0.6097 ± 0.0258 c	0.1230 ± 0.0053 bc	0.4613 ± 0.0247 de	0.00148 ± 0.00013 de	0.00089 ± 0.00007 abc
Source of variation							
Cultivar (CV)		***	***	***	***	***	NS
Temperature (T)		***	*	**	***	NS	***
CV × T		***	***	***	***	*	***

Means within a column, followed by different letters are significantly different at $p \leq 0.05$, with comparisons performed using Tukey's HSD test, $n = 4$. No letters denotes no significant differences between means. Levels of significance: * $p \leq 0.05$; ** $p \leq 0.01$; *** $p \leq 0.001$; NS = not significant. Each value represents the mean ± standard deviation. Cultivar/curd colour: Xenia F_1/white, Vitaverde F_1/green, Graffiti F_1/purple, Sunset F_1/orange, Celio F_1/romanesco. Temperature: 4 °C, transplant chilling; 18 °C, control.

Table 2S. Uptake of Sm, Sn, Sr, Ti, Yb, and Ce by cauliflower plants (mg per curds) as affected by cultivar and temperature treatment.

Cultivar	Temperature (° C)	Sm	Sn	Sr	Ti	Yb	Ce
Xenia F_1	4	0.0253 ± 0.0109 ab	0.0202 ± 0.0033 a	0.3446 ± 0.0087 a	0.1107 ± 0.0271 ab	0.00157 ± 0.00019 bcd	0.5227 ± 0.1504
	18	0.0149 ± 0.0055 ab	0.0233 ± 0.0082 a	0.3458 ± 0.0105 a	0.1062 ± 0.0200 abc	0.00307 ± 0.00109 a	0.5350 ± 0.3512
Vitaverde F_1	4	0.0342 ± 0.0163 ab	0.0161 ± 0.0043 ab	0.3557 ± 0.0180 a	0.1349 ± 0.0102 a	0.00074 ± 0.00033 bcd	0.1866 ± 0.0498
	18	0.0209 ± 0.0070 ab	0.0152 ± 0.0084 ab	0.3131 ± 0.0152 b	0.0764 ± 0.0110 cde	0.00108 ± 0.00049 bcd	0.3442 ± 0.1508
Graffiti F_1	4	0.0278 ± 0.0247 ab	0.0133 ± 0.0015 ab	0.2232 ± 0.0166 c	0.0857 ± 0.0067 bcd	0.00203 ± 0.00110 ab	0.3434 ± 0.1139
	18	0.0427 ± 0.0146 a	0.0044 ± 0.0017 b	0.1922 ± 0.0077 d	0.0833 ± 0.0108 bcd	0.00061 ± 0.00020 cd	0.3003 ± 0.1850
Sunset F_1	4	0.0226 ± 0.0088 ab	0.0153 ± 0.0063 ab	0.1645 ± 0.0050 e	0.0550 ± 0.0043 de	0.00078 ± 0.00033 bcd	0.2929 ± 0.0883
	18	0.0084 ± 0.0066 b	0.0119 ± 0.0008 ab	0.1309 ± 0.0033 f	0.0560 ± 0.0106 de	0.00055 ± 0.00027 d	0.2486 ± 0.1476
Celio F_1	4	0.0096 ± 0.0048 b	0.0107 ± 0.0063 ab	0.3135 ± 0.0050 b	0.0480 ± 0.0071 e	0.00201 ± 0.00055 abc	0.3636 ± 0.0449
	18	0.0166 ± 0.0091 ab	0.0158 ± 0.0055 ab	0.3073 ± 0.0126 b	0.0685 ± 0.0157 de	0.00162 ± 0.00034 bcd	0.3303 ± 0.1136
Source of variation							
Cultivar (CV)		**	***	***	***	***	*
Temperature (T)		NS	NS	***	NS	NS	NS
CV × T		NS	NS	**	***	***	NS

Means within a column, followed by different letters are significantly different at $p \leq 0.05$, with comparisons performed using Tukey's HSD test, $n = 4$. No letters denotes no significant differences between means. Levels of significance: * $p \leq 0.05$; ** $p \leq 0.01$; *** $p \leq 0.001$; NS = not significant. Each value represents the mean ± standard deviation. Cultivar/curd colour: Xenia F_1/white, Vitaverde F_1/green, Graffiti F_1/purple, Sunset F_1/orange, Celio F_1/romanesco. Temperature: 4 °C, transplant chilling; 18 °C, control.

Table 3S. Uptake of Dy, La, Sb, Tb, and Y by cauliflower plants (mg per curds) as affected by cultivar and temperature treatment.

Cultivar	Temperature (° C)	Dy	La	Sb	Tb	Y
Xenia F_1	4	0.00136 ± 0.00023 abc	0.00269 ± 0.00088 a	0.0799 ± 0.0117 ab	0.00130 ± 0.00047 ab	0.00117 ± 0.00062 b
	18	0.00151 ± 0.00030 ab	0.00184 ± 0.00029 bc	0.0809 ± 0.0142 ab	0.00165 ± 0.00035 ab	0.00376 ± 0.00153 a
Vitaverde F_1	4	0.00157 ± 0.00064 a	0.00206 ± 0.00029 ab	0.0970 ± 0.0238 a	0.00208 ± 0.00144 a	0.00120 ± 0.00090 b
	18	0.00085 ± 0.00020 bcd	0.00139 ± 0.00024 bcd	0.0598 ± 0.0246 abc	0.00124 ± 0.00036 ab	0.00136 ± 0.00023 b
Graffiti F_1	4	0.00078 ± 0.00022 cd	0.00114 ± 0.00012 cd	0.0430 ± 0.0083 bc	0.00083 ± 0.00065 ab	0.00130 ± 0.00060 b
	18	0.00102 ± 0.00010 abcd	0.00107 ± 0.00016 cd	0.0632 ± 0.0208 abc	0.00066 ± 0.00043 ab	0.00106 ± 0.00067 b
Sunset F_1	4	0.00081 ± 0.00015 cd	0.00096 ± 0.00033 d	0.0314 ± 0.0158 c	0.00094 ± 0.00031 ab	0.00110 ± 0.00030 b
	18	0.00052 ± 0.00013 d	0.00094 ± 0.00015 d	0.0458 ± 0.0172 bc	0.00039 ± 0.00023 b	0.00105 ± 0.00036 b
Celio F_1	4	0.00104 ± 0.00012 abcd	0.00113 ± 0.00009 cd	0.0386 ± 0.0161 bc	0.00143 ± 0.00040 ab	0.00055 ± 0.00006 b
	18	0.00139 ± 0.00031 abc	0.00125 ± 0.00019 bcd	0.0554 ± 0.0211 abc	0.00073 ± 0.00030 ab	0.00119 ± 0.00038 b
Source of variation						
Cultivar (CV)		***	***	***	**	***
Temperature (T)		NS	*	NS	NS	**
CV × T		**	*	*	NS	**

Means within a column, followed by different letters are significantly different at $p \leq 0.05$, with comparisons performed using Tukey's HSD test, $n = 4$. No letters denotes no significant differences between means. Levels of significance: * $p \leq 0.05$; ** $p \leq 0.01$; *** $p \leq 0.001$; NS = not significant. Each value represents the mean ± standard deviation. Cultivar/curd colour: Xenia F_1/white, Vitaverde F_1/green, Graffiti F_1/purple, Sunset F_1/orange, Celio F_1/romanesco. Temperature: 4 °C, transplant chilling; 18 °C, control.
